# Targeting pancreatic stellate cells in chronic pancreatitis: Focus on therapeutic drugs and natural compounds

**DOI:** 10.3389/fphar.2022.1042651

**Published:** 2022-10-19

**Authors:** Yang Wu, Chun Zhang, Mei Guo, Weikang Hu, Yangling Qiu, Mengran Li, Dong Xu, Pengfei Wu, Jing Sun, Run Shi, Zili Zhang, Kuirong Jiang

**Affiliations:** ^1^ Pancreas Center, The First Affiliated Hospital of Nanjing Medical University, Nanjing, China; ^2^ Gastroenterology Department, Jiangsu Province Hospital of Chinese Medicine, Affiliated Hospital of Nanjing University of Chinese Medicine, Nanjing, China; ^3^ Jiangsu Key Laboratory for Pharmacology and Safety Evaluation of Chinese Materia Medica, School of Pharmacy, Nanjing University of Chinese Medicine, Nanjing, China; ^4^ Department of Endocrinology, Jiangsu Province Hospital of Chinese Medicine, Affiliated Hospital of Nanjing University of Chinese Medicine, Nanjing, China; ^5^ Department of Oncology, The First Affiliated Hospital of Nanjing Medical University, Nanjing, China

**Keywords:** CP, PSCs, natural compounds, pancreatic fibrosis, anti-fibrotic drug

## Abstract

Chronic pancreatitis (CP) is a precancerous illness linked to pancreatic ductal adenocarcinoma (PDAC), although the evolutionary mechanism is uncertain. CP is distinguished by severe fibrosis caused by the activation of pancreatic stellate cells (PSCs). The current clinical therapeutic protocol for CP lacks specific therapeutic medicines for the prevention and suppression of inflammation and fibrosis aggravating in CP. More research on specifically targeting PSCs would help facilitate the development of novel therapies for pancreatic fibrosis. Notably, using natural compounds from medicinal plants as new antifibrotic agents has become a focus of recent research and is widely employed as an alternative and complementary approach. Our goal was to shed light on the role of PSCs in the development of CP and provide a focused update on the new potential therapeutic strategies against PSCs in CP models. Future studies can refer to these possible strategies for drug design, bioavailability, pharmacokinetics, and other issues to obtain better clinical outcomes for treating CP.

## Introduction

CP is characterized by inflammation, fibrosis, and loss of acinar and islet cells, which may present as abdominal pain, steatorrhea, pancreatic exocrine and endocrine insufficiency, and imaging-detectable pancreatic damage (Beyer et al., 2020). CP has a poorly known pathogenesis. Nonetheless, there appears to be a consistent sequence of an initial shock with damage, followed by recovery *via* fibrosis and regeneration. Recurrent oxidative stress or inflammation are fundamental causes of persistent pancreatic injury, causing irreversible changes in pancreatic structure and function and eventually leading to the onset of CP (Ren et al., 2020).

Pathogenesis of chronic pancreatitis differs based on underlying etiology, genetic background, and environmental exposures. Alcohol, the most prevalent cause of CP, may induce damage *via* toxic alcohol metabolites, overexpression of numerous genes related to cell death, direct stimulation of PSCs (resulting in fibrosis), and other mechanisms (Setiawan et al., 2017). Abnormal stromal/desmoplastic response is the hallmark histological feature of CP, which is attributed mainly to the activation of PSCs. Targeting PSCs may be a viable treatment strategy for CP (Kandikattu et al., 2020).

Currently, there are still few clinically available therapeutic agents for CP. Recently, some preclinical research into PSCs has been conducted, which will aid in creating innovative therapeutics for CP. In addition, natural chemicals derived from medicinal plants have great potential as antifibrotic drugs for the treatment of CP and deserve more in-depth studies *in vivo* and *in vitro* (Calfio et al., 2020).

This paper focuses on research targeting PSCs in CP models and reviews in detail the antifibrotic capabilities of several potential drugs for treating CP.

## Pancreatic stellate cells

Nearly a century after Karl von Kupffer’s discovery of hepatic stellate cells (HSCs) in 1876, Wateri’s group first detected similar cells (later termed PSCs) in the pancreas (Watari et al., 1982). It was not until 1998 that two milestone studies detailed the isolation and culture procedures of PSCs *in vitro*, allowing researchers to examine them in health and disease (Apte et al., 1998; Bachem et al., 1998). Since then, a slew of new research has been conducted in this area, and PSCs have become widely recognized as the primary source of the stromal/desmoplastic response that is characteristic of pancreatic cancer and CP (Apte and Wilson, 2004; Apte and Wilson, 2012; Pang et al., 2017).

PSCs have two phenotypes: quiescent (qPSCs) and activated (aPSCs). Under normal physiological conditions, qPSCs can be found in the peri-acinar or interlobular areas and comprise around 4% of the pancreatic cells (Apte et al., 1998; Bachem et al., 1998). qPSCs play a role in vitamin A storage, immunity, and the preservation of the normal structure in the pancreas (Allam et al., 2017). qPSCs have many typical features, such as abundant perinuclear lipid droplets, molecular markers (cytoglobin and adipophilin), and a low capacity to proliferate, migrate and synthesize ECM (Apte et al., 2013; Nielsen et al., 2017). Several factors can contribute to the activation process of qPSCs, such as smoking, alcohol intake, oxidative stress, hypoxia, etc. (Fu et al., 2018). In diseases like PDAC or CP, qPSCs get continuously activated and transform into aPSCs (Apte et al., 1999; Bynigeri et al., 2017). aPSCs show a myofibroblast-like phenotype: positive staining of α-smooth muscle actin (αSMA), loss of lipid droplets, increased production of cytokines and ECM (collagens, hyaluronic acid, fibronectin, etc.), and elevated ability to migrate, proliferate (Apte et al., 1999; Bynigeri et al., 2017). We have previously given a detailed overview of PDAC-associated PSCs, and the following will focus on their role in CP and targeted strategies (Wu et al., 2020).

## Pancreatic stellate cells in the development of chronic pancreatitis

**FIGURE 1:**
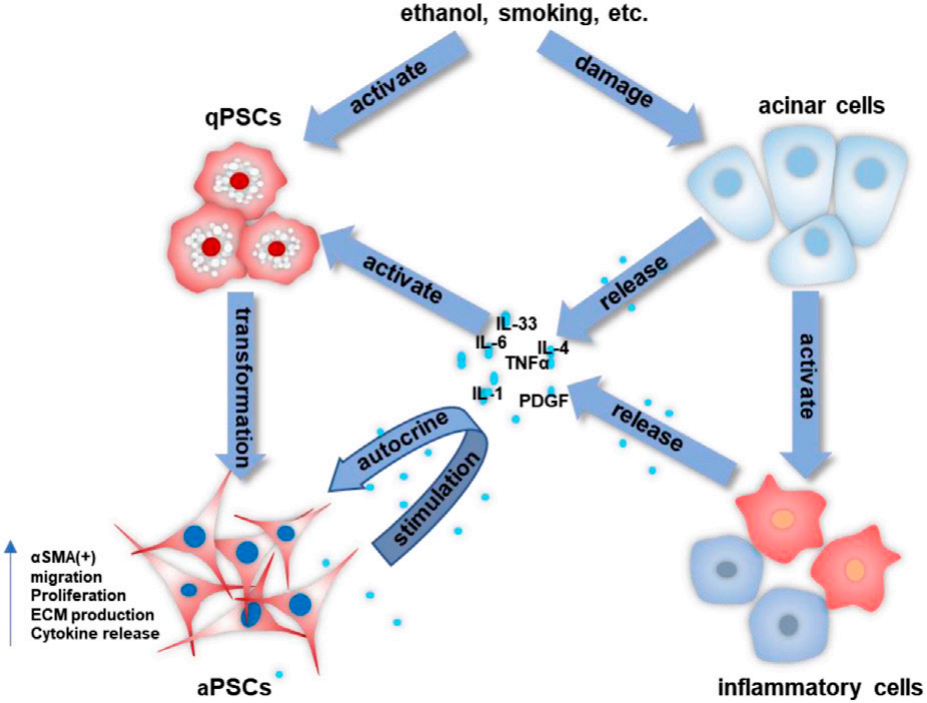
The activation process of PSCs in CP. The crosstalk among acinar cells, inflammatory cells, and stellate cells allows for the continued activation of PSCs and the progression of pancreatic fibrosis.

PSCs are special pancreatic resident cells that provide critical functions in both normal and abnormal pancreata. PSCs activation is a crucial step in the fibrogenic process of the pancreas (Bynigeri et al., 2017; Beyer et al., 2020). Multiple stimuli (ethanol, hyperglycemia, oxidative stress, cytokines, chemokines, etc.) can activate PSCs, causing them to release an abundance of ECM and hence promote the severe interlobular and intralobular desmoplastic reaction. Increased pancreatic fibrosis can lead to impaired exocrine and endocrine function of the pancreas. In early CP, damaged acinar cells stimulate major inflammatory cells (macrophages, granulocytes, and lymphocytes) that subsequently release large amounts of cytokines, such as interleukin (IL)-1/-4/-6/-8, tumor necrosis factor-alpha (TNF-α), transforming growth factor-beta 1 (TGF-β1), platelet-derived growth factor (PDGF), etc. (Jin et al., 2020; Kandikattu et al., 2020; Zheng et al., 2021). These molecules then stimulate PSCs, while PSCs can also secrete cytokines and continue to activate themselves through an autocrine pathway. The continuous stimulation of PSCs disrupts the synthesis-degradation balance of the ECM, ultimately leading to ECM deposition and fibrosis in the pancreas (Jin et al., 2020; Kandikattu et al., 2020; Zheng et al., 2021). Figure 1 is a schematic representation of the activation of PSCs in CP.

Several investigations on the mechanisms have been undertaken. Wu et al. indicated that TGF-β1 induced the activation of the nuclear factor kappa B (NF-κB) pathway in PSCs through modulating phospho-transforming growth factor beta-activated kinase 1 (p-TAK1), and this pathway might be a target for treating CP (Wu et al., 2021). Li et al. (2021a) showed that nicotine facilitated pancreatic fibrosis by inducing the activation of PSCs through Janus kinase 2/signal transducer and activator of transcription 3 (JAK2/STAT3) signaling pathway in rats, revealing the mechanism of smoking on CP. Lee et al. (2015) demonstrated that the cumulative effects of ethanol and cigarette components could activate PSCs in alcoholic smokers, accelerating the evolution of pancreatic fibrosis. Another study showed that the JAK/STAT pathway was essential for the proliferation and activation of PSCs and that inhibition of this pathway reduced caerulein-induced CP *in vivo* (Komar et al., 2017). Recently, Yang et al. indicated that very low-density lipoprotein receptor (VLDLR)-enhanced lipoprotein metabolism in PSCs increased fibrosis and that IL-33 played an essential role in this process in CP (Yang et al., 2022).

All these findings indicate that PSCs’ activation plays a crucial role in the development of CP, making PSCs a promising therapeutic target for CP.

## Drugs for the treatment of chronic pancreatitis by targeting pancreatic stellate cells

Even though the area of PSCs is still in its infancy, scientists have made significant efforts and proposed different strategies to target PSCs for treating CP. Recent research has indicated that siRNAs and small molecule kinase inhibitors can treat CP by targeting PSCs (Pezzilli et al., 2011; Tan et al., 2019; Mohanavelu et al., 2021). In addition, studies have shown that natural compounds from plants are widely used as complementary and alternative medications to treat diseases and extend life spans (Lin et al., 2015). Such drugs have promising clinical applications in the prevention and treatment of CP. The following is a discussion of the current status of several potential drugs and their possible mechanisms in treating CP.

Since morphological, functional, and gene expression investigations revealed considerable similarities between PSCs and HSCs, research on HSCs will be very informative (Erkan et al., 2012; Paulo et al., 2013). In addition, considering that PSCs derived from PDAC and CP have much in common in causing pancreatic fibrosis, some drugs of potential research value will also be listed here. Table 1 provides a summary of the existing antifibrotic strategies.

**TABLE 1:** Potential antifibrotic drugs in the treatment of CP.

Drugs	Diseases	Main results
Dasatinib	CP	Dasatinib inhibited the growth and activation of PSCs by inhibiting various TKs and MAPK cascades (Zeng et al., 2019).
Olaparib	CP	Olaparib showed antifibrotic effects in cerulein-induced CP (El-Hamoly et al., 2021).
Imatinib	CP	Imatinib reduced ECM deposition and PSCs’ activation while inhibiting the TGF-β1/Smad pathway (Bansod et al., 2021).
EGCG	CP	EGCG prevented fibrosis by decreasing PSCs’ activation through the antioxidative effect (Asaumi et al., 2006).
Emodin	CP	Emodin showed an antifibrotic effect on pancreatic fibrosis (Lin et al., 2015).
Curcumin	CP	Curcumin decreased the activation markers of PSCs (Lin et al., 2015).
Saikosaponin d (SSd)	CP	SSd decreased fibrosis by inhibiting the autophagy of PSCs *via* PI3K/Akt/mTOR signaling (Cui et al., 2019).
NLPR3 siRNA	CP	NLPR3 siRNA reduced the activation markers of PSCs (αSMA, collagen I, Fibronectin) (Li et al., 2021b).
YAP siRNA	CP	Activated YAP enhanced PSCs proliferation, and YAP siRNA decreased the activation of PSCs and fibrosis in CP (Spanehl et al., 2022).
Metformin	PDAC	Metformin reduced the cytokines released from cancer cells and inhibited the activation of PSCs in paracrine under co-culture (Duan et al., 2017).
Rhein	PDAC	Rhein inhibited fibrosis by decreasing the immunoreactivity of fibrotic activators and reducing fibronectin (Tsang and Bian, 2015).
Resveratrol	PDAC	Resveratrol suppressed ROS/miR-21-mediated activation and glycolysis in PSCs, inhibiting tumor invasion and migration (Yan et al., 2018).
DHA	Liver fibrosis	DHA alleviated liver fibrosis by triggering HSCs ferroptosis in an autophagy-dependent way (Shen et al., 2022).
EA	Liver fibrosis	EA exerted its antifibrotic effects by inducing FPN-dependent ferroptosis of HSCs by disrupting the formation of SNARE complexes (Li et al., 2022).
Angelica sinensis polysaccharide (ASP)	Liver fibrosis	ASP effectively alleviates chronic liver fibrosis by inhibiting HSCs activation through the IL-22/STAT3 pathway (Wang et al., 2020a).
Dendrobium officinale Polysaccharide (DOP)	Liver fibrosis	DOP maintained intestinal homeostasis by enhancing tight junctions between intestinal cells and reducing apoptosis, thereby inhibiting activation of the LPS-TLR4-NF-κB signaling pathway to protect against liver fibrosis (Wang et al., 2020b).
Celastrol	Liver fibrosis	Celastrol exerted anti-fibrotic effects by promoting ROS production and inducing ferroptosis in activated HSCs (Luo et al., 2022).

### siRNA

siRNAs represent a form of posttranscriptional gene silencing and have emerged as a fundamental and extensive regulator of gene expression. The most recent technology of high-throughput sequencing allows the analysis of siRNA abundance and expression profile in specific organs, tissues, and cells more and more precisely, allowing for the investigation of the roles of miRNAs and siRNAs (Fowler et al., 2018). mRNA transcripts of altered genes in genetic disorders and tumors are typical siRNA targets (Nambudiri and Widlund, 2013). Several studies have shown that the involvement of siRNAs in the expression of certain genes, which in turn influence the onset and progression of chronic pancreatitis, has potential therapeutic value (Charo et al., 2013). For instance, recent studies showed that the NACHT, LRR, and PYD domains-containing protein 3 (NLRP3) inflammatory bodies were directly involved in PSCs’ activation *in vivo* and *in vitro*. NLPR3 siRNA decreased the activation markers of PSCs (αSMA, collagen I, fibronectin) expression (Li et al., 2021b). Another study indicated that activated yes-associated protein (YAP) enhanced PSCs proliferation and knockdown of YAP by siRNA decreased the activation of PSCs and fibrosis in the CP animal model (Spanehl et al., 2022). However, these studies are still in the laboratory stage and must be further validated in clinical trials.

### Artemisinin and its derivatives

Artemisinin was identified as an isolated phytochemical derived from the Artemisia annua in 1972 (White et al., 2015). Several chemical derivatives have been developed to increase artemisinin’s water and oil solubility without compromising its therapeutic effect. Artemisinin was first used by the ancient Chinese to treat “fever,” commonly thought of as malaria (Miller and Su, 2011). This substance and its derivatives are still vital in treating malaria today.

Recent studies have also identified these chemicals’ role in treating different diseases, including inflammation, infection, cancer, and fibrosis (Efferth, 2006; Lai et al., 2013; Kim et al., 2015; Shi et al., 2015). Our preliminary research found that dihydroartemisinin (DHA) reduced liver fibrosis *via* inducing ferroptosis in HSCs (Shen et al., 2022). However, artemisinin and its derivatives have not been reported in pancreatic fibrosis in CP, which is a potential hot spot for future research.

### Vitamin A/D derivatives

In CP, vitamin A derivatives were reported to induce significant apoptosis, inhibit proliferation, and suppress ECM production of PSCs *in vitro*, indicating the potential of Vitamin A derivatives in reducing pancreatic fibrosis in CP (Xiao et al., 2015). However, the application of Vitamin A and its derivatives has been little studied in CP research. Therefore its use in targeting PSCs in PDAC is of a reference value. Previous research has demonstrated that Vitamin A analogs can inhibit the activation of PSCs in PDAC (Chronopoulos et al., 2016). ATRA (a Vitamin A analog) was found to reprise the quiescent state of PSCs in KPC mice (Froeling et al., 2011). All these studies show that Vitamin A analogs have potential research and clinical translational value in treating CP-related fibrosis.

A high prevalence of Vitamin D (VD) deficiency in CP patients is associated with the risk and prognosis of CP (Klapdor et al., 2012; Joker-Jensen et al., 2020). VD serves several biological roles in the body and has been extensively studied in inflammatory illnesses. VD and its derivatives have been found to inhibit PSCs’ activation and decrease ECM deposition, therefore relieving pancreatic fibrosis. Several investigations have shown that VD analogs may reprise the quiescence of PSCs and reduce fibrosis in CP and PDAC (Sherman et al., 2014; Wallbaum et al., 2018). These findings imply that VD might be an effective antifibrotic treatment for CP. More high-quality research and clinical trials are necessary to validate the anti-fibrosis function of VD in CP.

### Small molecule kinase inhibitors

Kinases have been the subject of contemporary pharmacological study due to their crucial function in cellular signal transmission (Wu et al., 2015). Previous research has shown that fibromodulin (FMOD) is increased in CP and is an essential downstream mediator of oxidative stress. The extracellular signal-regulated kinases (ERK) and c-Jun N-terminal kinases (JNK) inhibitors can decrease FMOD, thereby decreasing PSCs’ activation (An et al., 2020). Dasatinib, an inhibitor of several tyrosine kinases (TKs), was shown to have possible anti-fibrosis effects on CP and decrease pancreatic fibrosis and macrophage infiltration (Zeng et al., 2019). Olaparib, a peroxisome proliferator-activated receptor gamma (PARP) inhibitor, was reported to have antifibrotic effects in cerulein-induced CP (El-Hamoly et al., 2021). Imatinib, an inhibitor of discoidin domain receptor 1 (DDR1) and DDR2, can reduce ECM deposition and PSCs’ activation while inhibiting the TGF-β1/Smad pathway (Bansod et al., 2021). Small molecule kinase inhibitors are one of the current research hotspots and have promising applications in the treatment of CP.

### Resveratrol

Resveratrol is a polyphenolic stilbene in significant concentrations in grape, raspberry, blueberry, and peanut species (Rauf et al., 2018). Resveratrol has been shown to induce apoptosis of CP cells and alleviate fibrosis by promoting caspase-3 activation (Zhou et al., 2011). Previous studies have shown that resveratrol can suppress intracellular reactive oxygen species (ROS) and boost PSCs’ activation and glycolytic metabolism by suppressing miR-21 expression (Yan et al., 2018). Resveratrol inhibited ROS-induced activation of PSCs by decreasing miR-21 expression and raising the phosphatase and tensin homolog deleted on Chromosome 10 (PTEN) (Yan et al., 2018). Another study reported that resveratrol could inhibit the activation markers of PSCs (αSMA, collagen I, and fibronectin) by downregulating NF-κB signaling (Lin et al., 2015).

### Rhein

Rhein, the active component of rhubarb, is used extensively in treatment because of its anti-inflammatory, anti-angiogenic and anticancer characteristics (Xian et al., 2020; Li et al., 2021c). Rhein was reported to attenuate PSCs’ activation and suppress sonic hedgehog (SHH)/glioma-associated oncogene homolog 1 (GLI1) signaling in pancreatic fibrosis (Tsang et al., 2013). Rhein was able to suppress αSMA, Fibronectin, and matrix metalloproteinases (MMPs) in cultured PSCs *via* modulating the SHH pathway (Tsang et al., 2013; Tsang and Bian, 2015). Studies have shown that down-regulation of NF-κB and STAT3 signaling pathways may be the potential mechanism of Rhein’s anti-fibrosis and anti-tumor effects (Lin et al., 2015).

### Epigallocatechin gallate

Epigallocatechin gallate (EGCG), the main phenolic compound in green tea, was reported to have antioxidant, anti-inflammatory, and anticancer functions (Lee et al., 2014; Hosseini and Ghorbani, 2015). Polyphenols in green tea can prevent fibrosis by decreasing the activation of PSCs (Asaumi et al., 2006). Masamune et al. indicated that EGCG could reduce the ability of PSCs to proliferate and migrate (Masamune et al., 2005). *In vitro* experiments showed that EGCG pretreatment could inhibit the ethanol-induced activation of PSCs (Asaumi et al., 2007). Ethanol was known to increase the protein expression of αSMA, activate TGF-β1, and induce p38 mitogen-activated protein kinase (MAPK) phosphorylation. After EGCG treatment, p38 MAPK phosphorylation was eliminated, and the activation process of PSCs was inhibited (Asaumi et al., 2006).

### Metformin

Metformin has gained widespread use in treating type II diabetes. (Bailey, 2017). Studies have shown that metformin exerts anticancer effects by regulating inflammatory responses (Vancura et al., 2018). Previous studies have indicated that metformin significantly reduces the expression of αSMA and collagen and inhibits PSCs proliferation by upregulating phosphorylation-AMP-activated protein kinase (p-AMPK) expression (Duan et al., 2017).

### Emodin

Emodin, a natural anthraquinone derivative in some herbs, was reported to have anti-inflammation, anti-angiogenesis, anti-dyslipidemia, and anticancer functions (Gaman et al., 2018; Semwal et al., 2021). According to the literature, after 4 mmol/l emodin treatment, emodin downregulated the expression of several fibrosis markers (αSMA, fibronectin, and collagen I), thereby reducing the cell viability of primary PSCs (Lin et al., 2015). Table 1 lists the drugs that may be explored in CP treatment.

## Discussion

Multiple investigations have established that PSCs play a critical role in CP. All the above treatments have been reported to prevent the activation of PSCs in CP and the resulting fibrosis. These studies have also revealed the molecular mechanisms of the various drugs.

We address several drugs with antifibrotic effects that may serve as innovative antifibrotic treatments for PSCs in CP. Notably, phytochemicals should be encouraged for further in-depth research due to their cheap, non-toxic nature and sound therapeutic effects on PSCs in CP. Based on preclinical studies, given their potential as new antifibrotic drugs, it is important to consider drug delivery and low bioavailability. Recently, applications of nanoparticle systems have emerged in clinical practice related to drug delivery (Poilil Surendran et al., 2017; Sharma et al., 2017). We are confident that there will be relevant studies to test and validate these hypotheses and find new solutions for treating CP in the future.

Apart from these, some other promising options, such as ferroptosis inducers, have not been studied in CP. In recent years, ferroptosis has become a new hotspot to describe the regulatory form of cell death. Ferroptosis is closely related to tumors, degenerative diseases, ischemia-reperfusion injury, and cardiovascular diseases (Wei et al., 2020). Previous research showed that tripartite motif-containing protein 26 (TRIM26), a ferroptosis inducer, promoted ferroptosis in HSCs to inhibit liver fibrosis, which indicated that ferroptosis inducers might be potential anti-fibrosis agents (Zhu et al., 2021). Ellagic acid (EA), a natural product, exerted its antifibrotic effects by inducing ferroportin (FPN)-dependent ferroptosis of HSCs by disrupting the formation of SNARE complexes in liver fibrosis (Li et al., 2022). Luo et al. indicated that celastrol demonstrated anti-fibrotic effects on activated HSCs *via* boosting ROS generation and triggering ferroptosis (Luo et al., 2022). Our published paper has also revealed that DHA alleviates liver fibrosis by inducing ferroptosis in HSCs. We uncovered the possible mechanism of DHA against hepatic fibrosis and demonstrated that ferroptosis might be a novel method to remove activated HSCs (Zhang et al., 2021). Although these medications have not been investigated for CP fibrosis, their scientific value and clinical application possibilities in CP are promising.

However, drug research in CP still faces many challenges. When testing new treatments, it is vital to choose models that replicate the realities of CP in humans as closely as possible. There is still a long way to go in the development of new CP models. Besides, it is essential to note that the treatment modalities in these experimental models were performed at the same time as or before the induction of CP, which does not fully reflect the actual clinical situation. Therefore, the next major step is to construct phase I and subsequent trials for human CP so that the laboratory outcomes may be transferred to the bedside.
